# A Multimodality Machine Learning Approach to Differentiate Severe and Nonsevere COVID-19: Model Development and Validation

**DOI:** 10.2196/23948

**Published:** 2021-04-07

**Authors:** Yuanfang Chen, Liu Ouyang, Forrest S Bao, Qian Li, Lei Han, Hengdong Zhang, Baoli Zhu, Yaorong Ge, Patrick Robinson, Ming Xu, Jie Liu, Shi Chen

**Affiliations:** 1 Public Health Research Institute of Jiangsu Province Nanjing China; 2 Institute of HIV/AIDS/STI Prevention and Control Jiangsu Provincial Center for Disease Control and Prevention Nanjing China; 3 Department of Orthopaedics Union Hospital Huazhong University of Science and Technology Wuhan China; 4 Department of Computer Science Iowa State University Ames, IA United States; 5 Department of Pediatrics Affiliated Kunshan Hospital of Jiangsu University Kunshan China; 6 Department of Occupational Disease Prevention Jiangsu Provincial Center for Disease Control and Prevention Nanjing China; 7 School of Public health Nanjing Medical University Nanjing China; 8 Department of Software and Information Systems University of North Carolina at Charlotte Charlotte, NC United States; 9 Department of Public Health Sciences University of North Carolina at Charlotte Charlotte, NC United States; 10 Department of Radiology Union Hospital Huazhong University of Science and Technology Wuhan China; 11 School of Data Science University of North Carolina at Charlotte Charlotte, NC United States

**Keywords:** COVID-19, clinical type, multimodality, classification, machine learning, machine learning, diagnosis, prediction, reliable, decision support

## Abstract

**Background:**

Effectively and efficiently diagnosing patients who have COVID-19 with the accurate clinical type of the disease is essential to achieve optimal outcomes for the patients as well as to reduce the risk of overloading the health care system. Currently, severe and nonsevere COVID-19 types are differentiated by only a few features, which do not comprehensively characterize the complicated pathological, physiological, and immunological responses to SARS-CoV-2 infection in the different disease types. In addition, these type-defining features may not be readily testable at the time of diagnosis.

**Objective:**

In this study, we aimed to use a machine learning approach to understand COVID-19 more comprehensively, accurately differentiate severe and nonsevere COVID-19 clinical types based on multiple medical features, and provide reliable predictions of the clinical type of the disease.

**Methods:**

For this study, we recruited 214 confirmed patients with nonsevere COVID-19 and 148 patients with severe COVID-19. The clinical characteristics (26 features) and laboratory test results (26 features) upon admission were acquired as two input modalities. Exploratory analyses demonstrated that these features differed substantially between two clinical types. Machine learning random forest models based on all the features in each modality as well as on the top 5 features in each modality combined were developed and validated to differentiate COVID-19 clinical types.

**Results:**

Using clinical and laboratory results independently as input, the random forest models achieved >90% and >95% predictive accuracy, respectively. The importance scores of the input features were further evaluated, and the top 5 features from each modality were identified (age, hypertension, cardiovascular disease, gender, and diabetes for the clinical features modality, and dimerized plasmin fragment D, high sensitivity troponin I, absolute neutrophil count, interleukin 6, and lactate dehydrogenase for the laboratory testing modality, in descending order). Using these top 10 multimodal features as the only input instead of all 52 features combined, the random forest model was able to achieve 97% predictive accuracy.

**Conclusions:**

Our findings shed light on how the human body reacts to SARS-CoV-2 infection as a unit and provide insights on effectively evaluating the disease severity of patients with COVID-19 based on more common medical features when gold standard features are not available. We suggest that clinical information can be used as an initial screening tool for self-evaluation and triage, while laboratory test results should be applied when accuracy is the priority.

## Introduction

COVID-19 is a pandemic disease caused by the novel SARS-CoV-2 virus. As of January 12, 2021, COVID-19 had spread through at least 220 countries and regions, resulting in more than 88 million cases and almost 2 million deaths [1]. It has become the single most severe pandemic in the 21st century, dwarfing other coronavirus-caused epidemics, such as severe acute respiratory syndrome (SARS) in 2003 and Middle East respiratory syndrome (MERS) in 2012. COVID-19 is especially challenging to health professionals and the general population. Unlike in the preceding SARS and MERS epidemics, patients with COVID-19 can be either asymptomatic or symptomatic, and the virus has been demonstrated to be transmissible in both states to varying degrees [2-5]. In addition, the distinct clinical types of COVID-19, nonsevere and severe, require different treatment and care plans [6]. In current studies, patients with COVID-19 can be differentiated from patients who do not have the disease; however, further detection of nonsevere or severe types of COVID-19 has not been comprehensively explored. Patients with nonsevere COVID-19 can be accommodated with less intensive clinical monitoring and intervention, including treating pre-existing conditions and preventing health care–associated infections and other comorbidities [7]. In contrast, patients with severe disease require close monitoring, usually in the intensive care unit (ICU), by more clinicians [6]. Therefore, effectively and efficiently classifying clinical types of COVID-19 is essential for triage, resource optimization, and care planning for frontline clinicians and health care systems as well as for the patients [6,8].

Currently, nonsevere and severe COVID-19 types are classified based on only a few clinical features in China, including shortness of breath, O_2_ saturation, and PaO_2_ [9]. Because of the complexity of the pathological, physiological, and immunological response of COVID-19, these three features do not sufficiently characterize the difference between nonsevere and severe types in patients with COVID-19 [9-11]. Although shortness of breath can be self-monitored, O_2_ saturation and PaO_2_ cannot be accurately self-evaluated and may not be readily assessed in clinical settings, especially for socioeconomically disadvantaged patients. In addition, some patients with severe disease may not present shortness of breath initially. However, without proper medical intervention, their clinical course will worsen abruptly, often resulting in respiratory failure with high mortality [6]. Therefore, these gold standard features bear the risk of misclassification and misdiagnosis. Misclassification of COVID-19 clinical types can result in inappropriate early treatment decisions; this can place patients at risk of progression due to insufficiently aggressive supportive therapy or expose other patients to overly invasive treatment, both of which have negative clinical consequences. In addition, the three defining features may not be readily available during initial diagnosis when resources are inadequate.

It is therefore critical to provide a rapid, accurate, and efficient method to determine the severity of COVID-19 infection and identify the clinical type using alternative features. This determination will enable optimization of treatment plans for patient care and improve utilization of health care resources and staff. We suggest that additional readily available medical features, including the patient's comorbidities (eg, hypertension and diabetes) and symptoms (eg, fever and chest pain), as well as laboratory test results, can be used to develop an effective method to determine the clinical type and severity of COVID-19 [12,13]. Angiotensin-converting enzyme 2 (ACE-2) receptors, which facilitate SARS-CoV-2 infiltration, are distributed across multiple organs and systems in the human body [14]. More recent discoveries have found that in addition to the respiratory system, SARS-CoV-2 can invade digestive, reproductive, and even neural systems [15-18]. In other words, all clinical and laboratory test information of patients with COVID-19 could be consequences or risk factors of SARS-CoV-2 infection. In clinical practice to treat COVID-19, clinicians not from respiratory units or ICUs may rely only on the referenced features [9] while neglecting diverse and important clinical features of COVID-19, and they may miss critical signs leading to undesirable prognosis.

The potential power of clinical and laboratory testing features, as well as their combinations, to determine COVID-19 clinical type is currently being explored [19-24]. To use such diverse multimodality information as alternative evidence to facilitate accurate classifications, we propose a data mining and machine learning (ML) framework as an alternative to commonly used hypothesis-driven parametric models. The goal of this study is to provide reliable data-driven support for clinicians, even those who do not have comprehensive experience in diagnosing the emerging disease COVID-19. We aim to explore and contrast the distributions of clinical and laboratory testing features between nonsevere and severe COVID-19 types. We will identify key features that differ substantially between the two clinical types. Next, we will investigate whether a single modality or specific combination of features across modalities are able to provide accurate classification models via ML techniques. Specifically, we aim to identify a small and practical set of input features that can accurately differentiate COVID-19 clinical types. The insights gained from this study, as well as the developed end-to-end multimodal data analysis and ML framework, will enable us to better understand the comprehensive pathology of COVID-19, further distinguish COVID-19 from other respiratory infections, and apply the framework to other diseases with multimodal medical data in the future.

## Methods

### Data Source and Clinical Feature Extraction

In this study, we recruited 362 patients with COVID-19 from January to March 2020, including 148 patients presenting with severe disease and 214 patients lacking criteria for severe disease during admission, from Wuhan Union Hospital, China. The definitions of nonsevere and severe cases were mainly adopted from the official COVID-19 Diagnosis and Treatment Plan from the National Health Commission of China, and we also consulted guidelines from the American Thoracic Society [9-11]. Patients with severe COVID-19 should present any one of the following features: (1) respiratory rate >30 breaths per minute; (2) oxygen saturation <93% at rest; or (3) PaO_2_/fraction of inspired oxygen <300 mm Hg (40 kPa). Each patient with COVID-19 was confirmed by two independent quantitative reverse transcriptase–polymerase chain reaction tests before being included in this study. All patients or their responsible surrogates signed informed consent forms prior to study inclusion. The patients’ symptoms were evaluated and blood samples were drawn upon admission to perform laboratory testing. No pediatric patients aged less than 18 years were included.

The patients’ deidentified medical information include two major modalities of features, both of which were assessed at the time of admission. The first modality was a total of 26 pre-existing comorbidities and symptoms, referred to as “clinical features” hereinafter. These features included gender, age, hypertension, coughing, and different types of fever. A detailed description of these 26 features is provided in Table S1 in Multimedia Appendix 1. All clinical features were coded as 0-1 binary variables (age was dichotomized using 50 years as the threshold).

In addition, we collected the patients’ laboratory test results. The laboratory tests were plasma, serum, or whole blood assays for commonly obtained biochemistry tests, complete blood counts with differential counts and percentages, immunologic markers, such as interleukin 6 (IL-6), dimerized plasmin fragment D (D-dimer) and high-sensitivity C-reactive protein (hsCRP). After initial screening, several features with too many missing data, such as calcitonin, were excluded. In addition, respiratory rate, oxygen saturation, and PaO_2_ without supplemental oxygen were excluded because they are type-defining features according to the official National Diagnosis and Treatment Plan of China [9]. We used 26 laboratory test features in this study. Detailed descriptions and units of these features are provided in Table S2 in Multimedia Appendix 1. All these laboratory testing features were continuous features, in contrast to the binary features used in the clinical feature modality.

Patient-specific identifying information (eg, name and address of residence) was removed from the data collected for this study. This study was evaluated and approved by the IRB committee of Union Hospital, Wuhan, China (approval number: 2020-IEC-J-345).

### Data Mining on Multimodal Features

Initial data mining on the multimodal COVID-19 data was conducted. The patients’ clinical data were complete. Approximately 5% of the laboratory testing data were missing. Predictive mean matching (PMM) was applied to impute the missing data. To evaluate the effectiveness of PMM, we used a subset of the original data set with no data missing, randomly dropped 5% data to simulate potential data loss, re-extrapolated the data with PMM, and evaluated the root mean square error (RMSE) between the original and imputed data sets. The RMSE was less than 0.05, indicating that the extrapolation was feasible and reliable. The imputed data were then passed on to successive data mining and ML steps.

The prevalence of each clinical feature was calculated as the number of positive test results divided by the number of patients in the nonsevere and severe groups as defined by the Diagnosis and Treatment Plan [9]. The *z* test was applied to detect any statistically significant differences in the features between the two types. In addition, a forest plot of the odds ratios (ORs) and 95% confidence intervals of the clinical features between severe and nonsevere COVID-19 types was graphed.

For the continuous laboratory testing features, we characterized and contrasted the distribution of each feature between the two types. Because the values of most features were not normally distributed, we applied a 2-sided Kolmogorov-Smirnov test instead of the Student *t* test to determine whether distributions of the feature values differed significantly between the two clinical types.

### COVID-19 Clinical Type Classification via ML

Commonly used hypothesis-driven parametric models rely heavily on human decisions of how features interact with each other (eg, interaction terms in the logistic regression model), which may not reflect the underlying medical reality. In addition, these models have strict prerequisites to perform correctly, including normality of residuals, homoscedasticity, and independence of input features. Our initial exploratory analyses showed the that input features in both the clinical and laboratory testing modalities had nonnormality and high collinearity among the features. Another technical challenge to logistic regression in this study was the mixture of binary clinical and continuous laboratory testing input features.

Due to these problems, logistic regression would not be a preferred modeling approach to accurately classify and predict COVID-19 clinical types. Our exploratory analysis showed that logistic regression could only achieve average predictive accuracies of 68% and 77% on an 80-20 training-testing split using clinical and laboratory testing feature data sets, respectively (Table S3, Multimedia Appendix 1). Thus, logistic regression is less feasible in clinical settings, where high accuracy, sensitivity, and specificity are required to differentiate COVID-19 clinical types.

On the other hand, state-of-the-art ML classification models work directly with data to avoid possible human bias. In addition, ML models do not have restrictions on how input data should be distributed or related. Therefore, in this study, we determined that ML classification would be a more appropriate modeling approach to predict COVID-19 clinical type with a complicated data structure. We developed an end-to-end ML analytical framework to accurately predict the clinical type of patients with COVID-19 based on clinical and laboratory testing modality features. We built random forest (RF) classification models, as RF enables excellent interpretability of the relative importance of an input variable to provide a more comprehensive understanding of the pathobiology of COVID-19. RF is a widely used ML model based on decision theory and the decision tree approach. Due to the internal validation process with out-of-bag error measurement, RF is especially accurate and reliable. Unlike other commonly used ML models (eg, support vector machine or k-nearest neighbor), which usually require a separate cross-validation set, the RF model performs internal validation and is especially suitable when the data set is not large. In addition, RF is robust against data loss and data unbalancing (eg, there are more patients with nonsevere than severe disease in our study [25-29]). Because the major goal of this study was not to compare the performance of different ML models, we focused on RF to deliver the most accurate classification possible.

For the single modality RF model, we used 50% randomly selected data for both clinical and laboratory testing blood biochemistry features. In this step, 107 patients with nonsevere COVID-19 and 74 patients with severe COVID-19 were randomly chosen, while the other patients’ information was held to build the multimodal RF model. We assigned severe cases as “positive” and nonsevere cases as “negative” in the classification. The goal of ML classification through RF was to accurately predict the patient’s COVID-19 type, either positive (severe) or negative (nonsevere), based on features from different clinical modalities. In this part of the study, we first used a single modality of features, either clinical or laboratory testing, as the input. The detailed RF modeling and validation processes are provided in Multimedia Appendix 2. We trained the model with 100 independent runs; in each run, a different set of 80% of the data was randomly selected for training, while the remaining 20% of the data were held for testing only. This step was performed to explore whether the RF model was robust against different input data and to assess the generalizability of the model. Hyperparameters in this RF model include using Gini impurity to determine the decision tree split, a minimum of 2 samples for tree spit, a minimum of 1 sample at any leaf node, and a total of 8 trees for the model ensemble [26]. Important ML performance metrics, including accuracy, sensitivity, specificity, F1 score, and area under the curve (AUC) value based on the receiver operating characteristic (ROC) curve, were computed for the testing set only.

In addition, RF can evaluate the relative importance of the input variables based on their Gini importance scores [28]. We further quantified the Gini impurity importance scores of the input features in each RF run during the model development stage on the training set (80% randomly selected data). We then identified the top contributing features based on the Gini importance scores in each of the 100 runs, aggregated over 100 runs; identified the overall top contributing features; and explored the clinical relevance and interpretability of these features for COVID-19. Note that the Gini impurity importance was calculated from the RF model based on the training set only and not on the testing set. In addition, each run of the RF model was based on a completely different, randomly sampled, and independent set of 80% training data, from which the Gini importance was calculated. Therefore, this approach avoided potential issues of overfitting and inflated performance [30]. If an RF model is robust, important input features should be consistent with the different 80% portions of the data used as the training set to develop the model. The most important features to differentiate COVID-19 clinical types were also cross-checked with our results from exploratory data mining, including the prevalence of the clinical features and the distribution of the laboratory testing features.

### COVID-19 Clinical Type Classification With Multimodal ML

More importantly, we explored whether and how combining features across feature modalities improved classification performance. We developed another RF model with the same hyperparameter setting that incorporated features from both modalities. The modeling process using a single modality was similar. Instead of putting all 52 features into the model, we selected only the top 5 features from each of the two modalities as new inputs to reduce the increase model feasibility in case certain features in the total 52-feature pool would not be readily available. These top features were identified from the Gini importance of the single modality RF models (highlighted in Table S1 and Table S2 in Multimedia Appendix 1). The data set for developing the multimodal RF model was a completely new data set, as in, the other 50% of the original data was based on 107 additional patients with nonsevere COVID-19 and 74 additional patients with severe COVID-19 whose data were not used in the development of the single modality RF model.

We explored whether only 10 important features from different modalities could perform sufficiently well to address the clinical challenge of differentiating COVID-19 clinical types. This study can serve as alternative and supplemental tool to the gold standard features, which may not be readily available at the time of diagnosis.

All statistical analyses and ML models were built in R 4.0.2 (R Project) and Python 3.7 with additional supporting packages. The complete codes and fully deidentified data are freely available on GitHub [31].

## Results

### Clinical Findings in Nonsevere and Severe COVID-19

**Figure 1:**
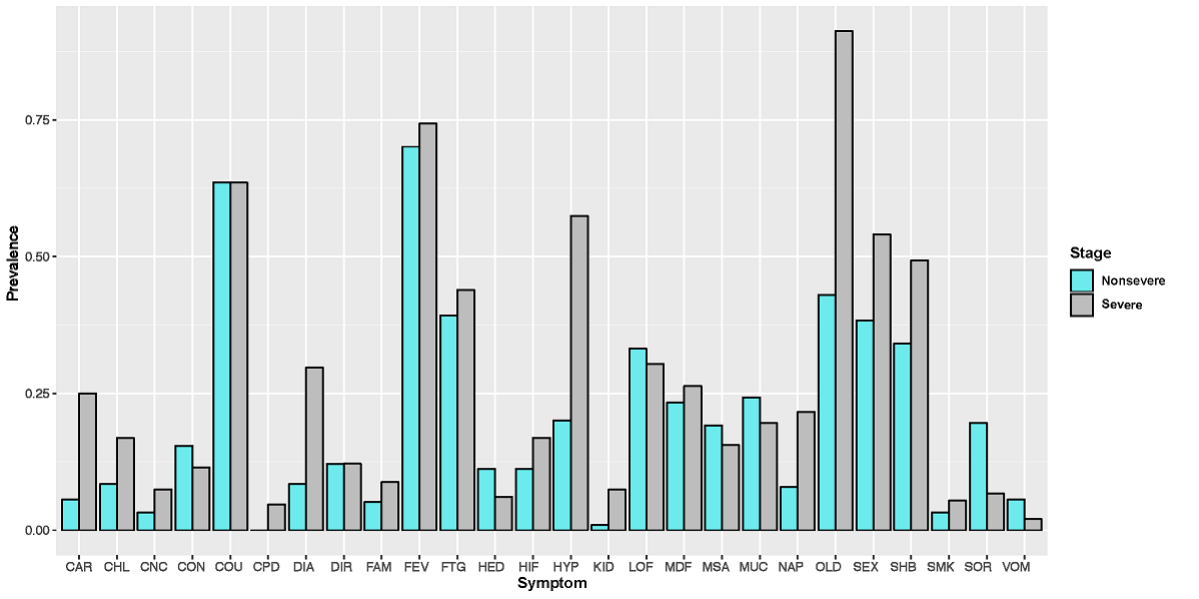
Comparison of clinical features of patients with nonsevere and severe COVID-19. Note that because these features were binary, the y-axis indicates the prevalence of a positive result. CAR: cardiovascular disease; CHL: chills and shaking; CNC: cancer; CON: contact with patients with COVID-19; COU: coughing; CPD: chronic obstructive pulmonary disease; DIA: diabetes; DIR: diarrhea; FAM: family members with COVID-19; FEV: fever; FTG: fatigue; HED: headache; HIF: high fever; HYP: hypertension; KID: renal disease; LOF: low fever; MOF: medium fever; MSA: muscle ache; MUC: phlegm; NAP: loss of appetite; OLD: older age; PREV: prevalence; SEX: male sex; SHB: chest congestion; SMK: history of smoking; SOR: sore throat; VOM: vomiting.

**Figure 2:**
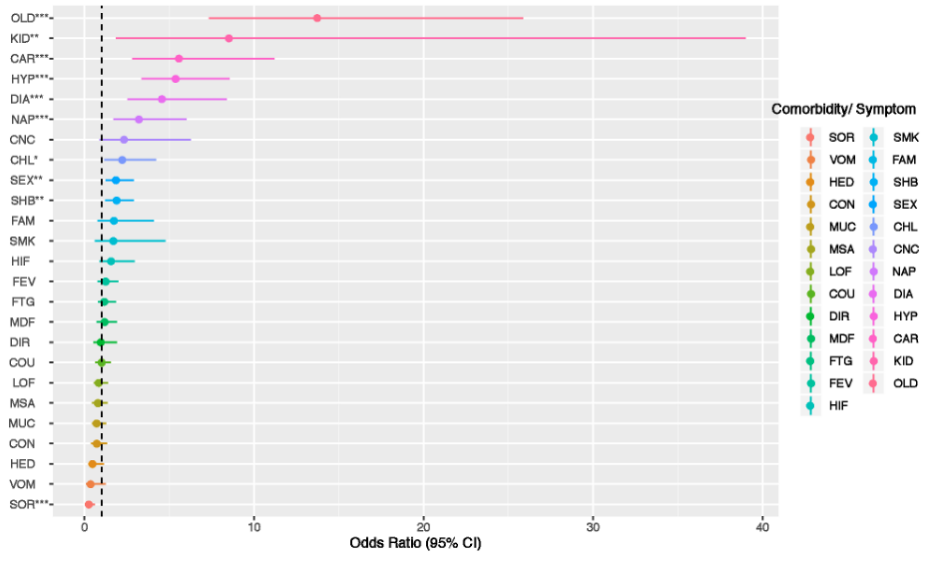
Forest plot of the importance of clinical features of patients with nonsevere and severe clinical types of COVID-19. Chronic obstructive pulmonary disease is not included because only patients with severe COVID-19 showed it as a comorbidity. The threshold for a feature to be positively or negatively associated with severe COVID-19 was 1 (dashed line), not 0. CAR: cardiovascular disease; CHL: chills and shaking; CNC: cancer; CON: contact with patients with COVID-19; COU: coughing; CPD: chronic obstructive pulmonary disease; DIA: diabetes; DIR: diarrhea; FAM: family members with COVID-19; FEV: fever; FTG: fatigue; HED: headache; HIF: high fever; HYP: hypertension; KID: renal disease; LOF: low fever; MOF: medium fever; MSA: muscle ache; MUC: phlegm; NAP: loss of appetite; OLD: older age; SEX: male sex; SHB: chest congestion; SMK: history of smoking; SOR: sore throat; VOM: vomiting. *P<.05, **P<.01; ***P<.001 from the 2×2 contingency table for each feature.

The prevalence of clinical features in patients with nonsevere and severe COVID-19 at the time of entry into the study were calculated and compared (Figure 1). A more detailed comparison of the clinical features between the two COVID-19 clinical types is provided in Table S1 in Multimedia Appendix 1, which shows the ORs, confidence intervals, and associated *P* values. For patients with the two clinical types of COVID-19, the prevalence was distinct for a number of different features. Patients with severe COVID-19 were statistically much more likely to be older (aged ≥50 years, OR 13.77, 95% CI 7.33-25.86, *P*<.001) and male (OR 1.89, 95% CI 1.24-2.90, *P*=.003) and to have renal diseases (OR 8.51, 95% CI 1.86-38.99, *P*<.001), cardiovascular diseases (OR 5.61, 95% CI 2.81-11.20, *P*<.001), hypertension (OR 5.37, 95% CI 3.36-8.56, *P*<.001), diabetes (OR 4.61, 95% CI 2.53-8.38, *P*<.001), loss of appetite and taste (OR 3.20, 95% CI 1.70-6.01, *P*<.001), chills (OR 2.21, 95% CI 1.16-4.22, *P*=.01), and chest congestion (OR 1.88, 95% CI 1.22-2.89, *P*=.003) than their counterparts with nonsevere COVID-19. The only exception was sore throat, which patients with severe COVID-19 were significantly much less likely to develop (OR 0.30, 95% CI 0.14-0.61, *P*<.001). These discoveries are further demonstrated in the forest plot of the ORs and confidence intervals in Figure 2, which shows the differences between the two clinical types. Therefore, these relatively easily measured and acquired clinical features could be used to clinically evaluate the disease severity of patients with COVID-19. Our findings, especially for patients with severe COVID-19, echoed the US Centers for Disease Control and Prevention’s recently updated list of symptoms of COVID-19 [32] and more recent characterizations of patients with COVID-19 in the United States [33]. Our findings showed that older male patients with COVID-19 who had cardiovascular disease, respiratory disease, renal disease, and diabetes were at much higher risk of developing serious complications of COVID-19, such as acute respiratory distress syndrome (ARDS) and even death [20,21]. In addition, we discovered that Chinese patients with renal diseases were significantly more likely to develop severe COVID-19, which has not been widely reported. Clinical evidence has shown that ACE-2 expression is associated with kidney diseases; thus, kidney disease is a potential complication of SARS-CoV-2 infection [34,35]. This finding would inform clinicians that they should also monitor kidney dysfunction, such as acute kidney injury, as a clinical sign or consequence of severe COVID-19 complications.

**Figure 3:**
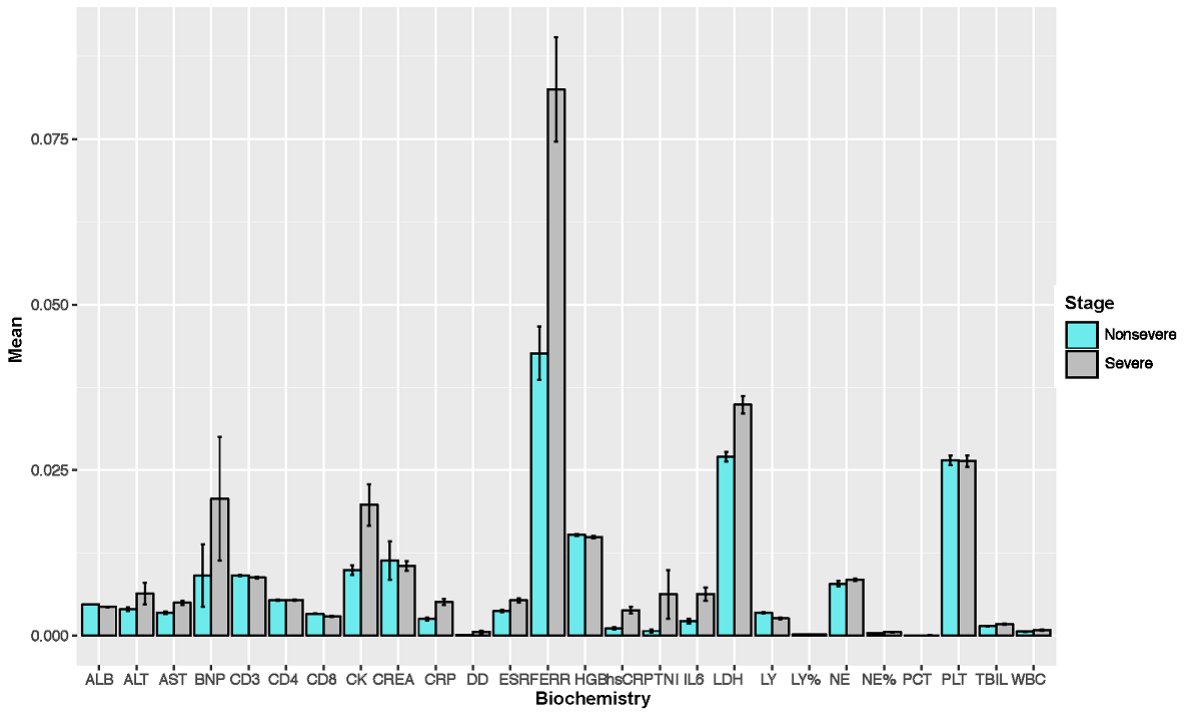
Comparison of laboratory testing features of patients with nonsevere and severe COVID-19. Values shown on the y-axis were obtained after feature scaling and are between 0 and 1. The error bars represent the standard error of each laboratory testing feature. ALB: albumin; ALT: alanine transaminase; AST: aspartate aminotransferase; BNP: Brain natriuretic peptide; CK: creatine kinase; CREA: creatinine; CRP: C-reactive protein; DD: dimerized plasmin fragment D; ESR: erythrocyte sedimentation rate; FERR: ferritin; HGB: hemoglobin; hsCRP: high-sensitivity C-reactive protein; TNI: troponin I; IL6: interleukin 6; LDH: lactate dehydrogenase; LY: lymphocyte; LY%: percent of lymphocytes; NE: neutrophil; NE% percent of neutrophils; PCT: procalcitonin; PLT: platelet; TBIL: total bilirubin; WBC: white blood cell.

For the laboratory testing modality features, we compared the distributions of the continuous features between nonsevere and severe COVID-19. The results are demonstrated in Figure 3. A more detailed comparison of these 26 laboratory testing features between the two clinical types is provided in Table S2 in Multimedia Appendix 1, which shows the *P* values from the Kolmogorov-Smirnov tests. Based on the 2-sided Kolmogorov-Smirnov test, severe and nonsevere COVID-19 types differed significantly in most laboratory features, except for platelet (PLT), hemoglobin (HGB), CD3, and CD4. Among all laboratory features, IL-6, high-sensitivity troponin I (hsTNI), and D-dimer had the most significant differences between nonsevere and severe COVID-19 types.

In conclusion, after extensive clinical feature extraction and data mining, we obtained strong qualitative and quantitative evidence that nonsevere and severe COVID-19 types differ substantially with regard to clinical features and laboratory test results. These findings pave the way toward creating an effective ML classifier to accurately differentiate these two COVID-19 types in clinical practice.

### Clinical Type Classification via ML

#### Comorbidity and Symptom (Clinical) Modality

**Figure 4:**
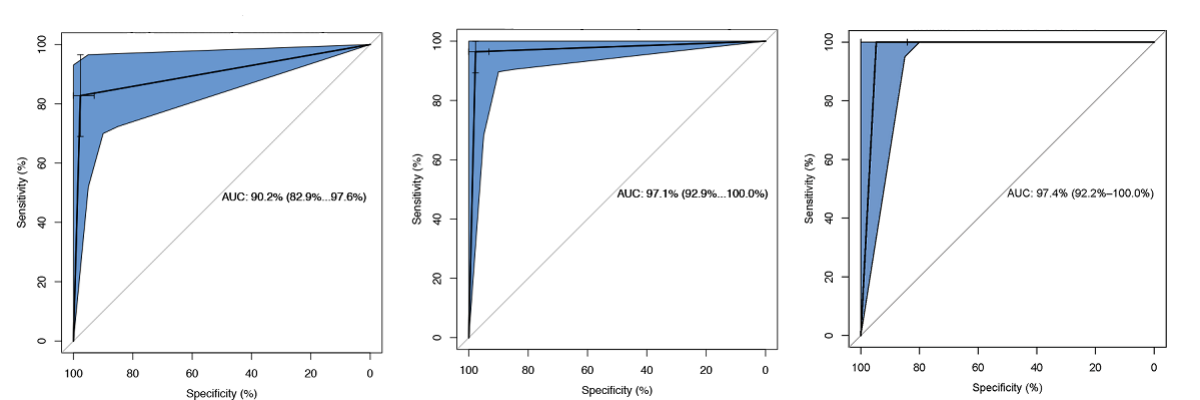
ROC curves from the random forest models based on clinical, laboratory testing, and multimodal features. Left: the symptom feature as the sole input; middle: the laboratory testing feature as the sole input; right: both features combined as the input. AUC: area under the curve; ROC: receiving operator characteristic.

We first explored whether relatively simple binary features could provide accurate insights in identifying COVID-19 disease severity. The performance of this model is summarized in the upper section of Table 1. Based on 100 independent runs, the RF model reached a median of >99% and 94% accuracy for the training and testing sets, respectively (Table 1). Median is reported instead of mean value because the performance metrics were not normally distributed. The AUC was 90.2% (range 82.9%-97.6%) based on the ROC curve (Figure 4, left panel). The model performed better in detecting true positives (ie, severe clinical type) than true negatives (ie, nonsevere type). In other words, clinical features alone in the RF models were very unlikely to misclassify a severe case as a nonsevere case but had a higher likelihood of predicting a nonsevere case to be a severe case. In clinical practice, this would be a lesser concern, as a false positive (failure to detect nonsevere type) would be more tolerable than a false negative (failure to detect severe type).

**Table 1:** Performance of the random forest model with multimodal features. The results are based on 100 runs. In each run, 80% of the data was randomly selected as the training set and 20% as the testing set. The table shows the model performance on the testing set only.

Feature and performance metric		Median	Minimum	Maximum
**Clinical (%)**				
	Accuracy	94.59	81.08	>99
	Sensitivity	>99	80.95	>99
	Specificity	93.75	75.00	>99
	F1 score	97.30	82.93	>99
	AUC^a^	90.20	82.90	97.60
**Laboratory testing (%)**				
	Accuracy	97.22	93.06	>99
	Sensitivity	>99	94.59	>99
	Specificity	96.97	83.33	>99
	F1 score	97.89	94.74	>99
	AUC	97.10	92.90	>99
**Multimodal (%)**				
	Accuracy	97.22	91.67	>99
	Sensitivity	>99	90.00	>99
	Specificity	94.44	75.00	>99
	F1 score	97.78	97.22	>99
	AUC	97.40	92.20	>99

^a^AUC: area under the curve

Our RF model also identified the major influential features to differentiate COVID-19 types based on their contributions to the Gini importance in the training set. The top influential clinical features, in descending order, were age, gender, hypertension, diabetes, and cardiovascular diseases, in accordance with existing literature reports [36]. Other important clinical features included fatigue, chest congestion, sore throat, phlegm, and fever. Most of these findings aligned well with our parametric data mining with OR comparison (Figure 2, Table S1 in Multimedia Appendix 1) but showed much higher accuracy (94% accuracy on the testing set of the RF model compared to 68% accuracy from logistic regression). The only exception was renal disease, which was not considered to be a major differentiating factor based on its Gini importance (Table S1, Multimedia Appendix 1).

Clinically, older male patients with pre-existing comorbidities, especially hypertension, diabetes, and cardiovascular diseases, are much more vulnerable to COVID-19 and have a much higher risk of developing severe disease [19,21]. Therefore, we suggested using the comorbidity and symptom features of patients with COVID-19 as the first round of evaluation of severity with reasonable accuracy.

#### Laboratory Testing Modality

The RF model with 26 laboratory testing features was highly effective in differentiating nonsevere and severe COVID-19. The RF model achieved >99% and >95% accuracy for the training and testing data sets, respectively. The sensitivity, specificity, and F1 scores were all >95% when using only 8 trees in the RF model (Table 1, middle section). The AUC was 97% based on the ROC curve (Figure 4, middle panel). Although this study focused on ML methods, we evaluated the model performance of non-ML logistic regression in Table S3 (Multimedia Appendix 1) as a reference point to show the improvement that state-of-the-art ML models could achieve.

The top differentiating features in the laboratory testing modality were D-dimer, hsTNI, neutrophil, IL-6, lactate dehydrogenase (LDH), and hsCRP, in descending order. The clinical interpretation of their important roles was that patients with severe COVID-19 experience more intense immune responses and hyperinflammation, such as cytokine storm syndrome, with substantially increased IL-6 [37]. Research has also shown that SARS-CoV-2 can infect many organs other than the lungs, including the heart, and induce dysfunction of these organs [38,39]. Increasing hsTNI was found to be a sign of heart tissue damage from SARS-CoV-2 infection [40]. In addition, patients with severe COVID-19 may have microthrombosis, which induces higher D-dimer levels [19,21,41-43]. Abnormal levels of neutrophils may be responsible for cytokine storms and ARDS in patients with severe COVID-19 [13,44]. hsCRP, a biomarker of acute inflammation, cardiovascular disease, and ischemic events, was also confirmed to be a major contributing factor of COVID-19 mortality [19]. LDH is a biomarker of tissue damage and has been used to predict the clinical course of patients with COVID-19 [45]. These findings add further clinical insights to how multiple organs and systems, not just the lungs, respond to SARS-CoV-2 infection in different clinical types [14,46,47].

#### Multimodal Features

We further developed a multimodal RF model that incorporated both clinical and laboratory testing modalities with a completely new data set that was not used for the single modality model development. We used only the 5 most important features from the clinical and laboratory testing modalities, based on their Gini importance scores. The results showed that the top 10 of a total of 52 features from both modalities achieved almost >95% in every model performance metric, including accuracy, sensitivity, specificity, and F1 score (Table 1). The AUC was >97% as well (Figure 4, right panel). Therefore, we concluded that a two-step evaluation and triaging process would be feasible to differentiate the clinical types of patients with COVID-19 when the gold standard type-defining features were not readily available.

These findings reflect our clinical understanding that SARS-CoV-2 attacks multiple organs and systems, and the human body reacts in a unity against infection. Different features (eg, comorbidity, symptom, and laboratory testing results) complemented each other to provide a more comprehensive characterization of how the human body as a united entity, not only the respiratory system, reacted to SARS-CoV-2 infection [14]. In addition, the decent model performance supports the feasibility of multimodal data mining in detecting and differentiating patients with nonsevere COVID-19 from patients with severe disease.

Comparing the original 52 features in both modalities, which may not be all available at the same time during COVID-19 diagnosis, the top 10 most differentiating multimodal features provided a more practical input combined with the highly accurate ML model. Therefore, we concluded that our work would help effectively optimize health care operations during the pandemic and avoid overloading of the health care system [8].

## Discussion

### Principal Findings

This study provides a novel analytical framework that combines the power of multiple clinical features from different modalities to differentiate COVID-19 clinical types via ML techniques. Practically, it enables the delivery of a more comprehensive understanding of the pathobiology of COVID-19. It can aid the development of optimal treatment plans for individual patients, such as sending them to a mobile cabin hospital or admitting to a hospital with an ICU [7]. In addition, it will enable more effective triaging and optimization of health care system resources and personnel. This will substantially reduce the risk of overloading the health care system by admitting all patients with COVID-19 to hospital, decrease potential health care–associated infections, and improve clinical outcomes for the patients, especially during the COVID-19 pandemic [8].

In addition to accurately detecting vulnerable patients with COVID-19 who are likely to have severe disease, this study also provides insights on why these patients may have severe disease. ML models work directly with data and therefore are generally not good at providing clear interpretations. In this study, we combined the power of both hypothesis-driven and data-driven ML models. The highest-contributing comorbidities, symptoms, and biochemical features help predict and explain potential COVID-19 clinical courses and prognoses. Our research echoes recent studies that characterize and predict the clinical course, critical illness, and mortality of patients with COVID-19 [13,19,21]. In particular, another decision tree–based algorithm, extreme gradient boosting (XGBoost), showed promising performance in predicting the mortality of patients with COVID-19 [19]. RF is technically similar to XGBoost, and our results were consistent in identifying the key differentiating features, including LDH and hsCRP.

A continuous-valued risk score calculator for predicting risk of transitioning to critical-type COVID-19 (an even more severe type that requires ICU hospitalization, an invasive ventilator, or extracorporeal membrane oxygenation, and has a mortality rate as high as 50%) has been developed for patients with COVID-19 [21]. As a comparison, although our RF model predicts a 0-1 binary outcome for nonsevere and severe type disease, the internal RF modeling process through decision tree approach actually calculates an intermediate score between 0 and 1. By using a cutoff threshold, the RF model reports a final dichotomized 0-1 outcome. Therefore, our analytical framework can also be readily adjusted to provide a continuous risk score for clinical evaluation and triaging of patients with COVID-19, if needed.

Many patients with severe COVID-19 present symptoms in lungs, especially ground-glass opacity (GGO), which can be detected by biomedical imaging techniques such as computed tomography (CT). However, a major clinical challenge of COVID-19 lies in the asymptomatic patient problem, which creates far worse difficulties than other coronavirus epidemics, including the original SARS and MERS epidemics. These patients show few or no classic symptoms related to viral pneumonia, and they present no GGO; however, they are almost as capable of transmitting the virus as symptomatic patients [4-6]. We suggest that the term “asymptomatic” is used due to lack of a comprehensive characterization and understanding of this novel pathogen and the pathophysiology of the host; we also suggest that these patients are not truly “asymptomatic,” as in, without any clinical symptoms or signs. Approximately 10% of the patients with mild COVID-19 in our study cohort did not show typical respiratory symptoms, including fever, coughing, and chest pain, upon admission. However, they showed other symptoms from the more comprehensive modality of 26 clinical features.

### Future Work

The next step of this study is to further include a biomedical imaging modality. A technical barrier is that a CT scan is a high-dimensional feature set, while clinical and laboratory test data have relatively low dimensionality. Therefore, the CT scan, in its original form of imaging, cannot be effectively combined with other modalities. We will evaluate the feasibility of using a convolution neural network (CNN, another ML technique) first to reduce the feature space in CT scans and extract a fully connected layer in the CNN as a representation of the CT scan feature. A fully connected layer is a 1D vector and has the same dimensionality as the other two modalities. Therefore, in theory, we would be able to further combine CT scans with other clinical features and investigate the association between these features with regard to COVID-19.

COVID-19 is a complex disease in which the pathogen not only attacks the respiratory system but other organs and systems that possess ACE-2 receptors as well [14,33]. Our findings reveal the complicated pathological, physiological, and immunological responses to SARS-CoV-2 infection and shed light in understanding the complex interactions between the virus and the human body. Although our multimodal data mining and ML framework was developed with data from patients with severe and nonsevere COVID-19, we suggest that the end-to-end framework is applicable to many disease systems in which multimodal inputs are common, including demographic information, comorbidity, laboratory testing, imaging, and -omics data. Having a more holistic viewpoint and approach will enable us to understand and respond to these emerging diseases, especially the unprecedented COVID-19, more readily in the field. Another feasible analytical solution is ensembling. Each input feature modality can be used independently to train a specific model, and the final prediction of COVID-19 clinical type can be made through ensembling. We will further explore this analytical framework and transfer our insights to future clinical studies, such as differentiating healthy patients from patients with non–COVID-19 viral pneumonia, nonsevere COVID-19, and severe COVID-19.

### Limitations

In this study, we recruited participants from a single hospital in Wuhan, the first epicenter of COVID-19. There will inevitably be selection bias, as the ethnic group is currently limited to Chinese participants who are mostly of Han ethnicity. It is possible that ethnicity and race and their confounding risk factors (eg, socioeconomic status, nutrition conditions, accessibility of care, and other social determinants of health) are different in various studies. Therefore, we wish to share our findings with our colleagues worldwide and determine whether different demographic backgrounds influence feature distributions between nonsevere and severe COVID-19 in patients. Some of our findings of the top contributing clinical and laboratory testing features were supported in other COVID-19 studies across different ethnic groups, while others were not [32,40]. For example, while we found male gender to be a strong influencing factor of severe COVID-19, other studies did not reach a similar conclusion [36]. The findings in this study on Chinese ethnicity could actually complement other existing studies on other ethnic groups and reveal the clinical and epidemiological complexity of this unprecedented ongoing pandemic.

Another limitation of this study is that the patients were evaluated at the time of admission; therefore, the study was a cross-sectional instead of a longitudinal study. Future studies could examine both diagnosis and prognosis and further explore how and why some patients with nonsevere COVID-19 may transition to a severe disease state and whether ML techniques are able to identify critical predictive features to undesirable prognoses such as death.

Additionally, different subtypes of SARS-CoV-2, their specific pathogenicity and virulence, and their host-pathogen interactions should be taken into consideration when conducting and comparing studies across different regions of the world. The other factors that this study did not include are behavioral and societal aspects, such as whether and how using mobile cabin hospitals to treat patients with nonsevere COVID-19 reduces the rate of transition to severe type. The COVID-19 epidemic, like all infectious disease epidemics, has individual clinical, epidemiological, behavioral and societal factors. Therefore, we will also explore cross-scale individual clinical course and population-level epidemics in future studies.

### Conclusion

We trained and validated ML RF models to predict COVID-19 severity based on 26 comorbidity and symptom features and 26 laboratory testing features from a cohort of 214 patients with nonsevere COVID-19 and 148 patients with severe COVID-19. We identified the top features from both feature modalities to differentiate the clinical types, and we achieved predictive accuracies of >90%, >95%, and >99% when clinical features, laboratory test data, and the top 5 features from each modality combined were used as inputs, respectively. The results will help patients with COVID-19 self-evaluate their condition, help clinicians to evaluate disease severity and triage patients, and optimize health care resource utilization during the COVID-19 pandemic.

## Appendix

Multimedia Appendix 1 Supplementary tables S1-S3.

Multimedia Appendix 2 Supplementary information regarding the model development and complete feature distributions.

## Abbreviations

ACE-2: angiotensin-converting enzyme 2
ARDS: acute respiratory distress syndrome
AUC: area under the curve
CT: computed tomography
D-dimer: dimerized plasmin fragment D
GGO: ground-glass opacity
hsCRP: high-sensitivity C-reactive protein
hsTNI: high-sensitivity troponin I
ICU: intensive care unit
IL-6: interleukin 6
LDH: lactate dehydrogenase
MERS: Middle East respiratory syndrome
ML: machine learning
OR: odds ratio
PMM: predictive mean matching
RF: random forest
RMSE: root mean square error
ROC: receiver operating characteristic
SARS: secure acute respiratory syndrome
XGBoost: extreme gradient boosting
