# Supplementary material for: A Multimodality Machine Learning Approach to Differentiate Severe and Nonsevere COVID-19: Model Development and Validation
Source: J Med Internet Res. 2021 Apr 7;23(4):e23948. doi: 10.2196/23948 (PMC8030658; doi:10.2196/23948)
Supplement: Multimedia Appendix 1 [file jmir_v23i4e23948_app1.docx]

**Supplementary Method: Random Forest (RF) Machine Learning Model Formulation**

In each run, we randomly selected 80% of all data as the training set to train the RF model (Fig. S1). These 80% data included both severe and non-severe cases, i.e., both positives and negatives. We also ensured that the distributions of positives and negatives in the training set was similar to those in the complete data. Once the model was developed, the remaining 20% data would be fed into the developed model to evaluate its performance on unseen testing data. This prediction process was crucial to ensure that the ML model was not over-fitting, i.e., the model worked extremely well on existing training data but poorly on unseen real-world data. We then constructed the 2x2 confusion matrix to evaluate the model performance on testing data. The 2x2 confusion matrix had four elements, true positive (TP, model correctly identified severe type), true negative (TN, model correctly identified non-severe type), false positive (FP, model incorrectly identified non-severe type as severe type), and false negative (FN, model incorrectly identified severe type as non-severe). Then, important ML model performance metrics were computed, including model accuracy, sensitivity, specificity, and F1 score, etc. Among these performance metrics, accuracy and F1 score evaluated overall performance of the model, sensitivity (also known as true negative rate, TNR) emphasized FN, and specificity (also known as true positive rate, TPR) emphasized FP. Our RF model aimed to increase TP and TN while simultaneously reducing FP and FN. In the other words, an ideal ML model should have both high sensitivity and high specificity. The highest possible value for these metrics was 1 (100%), indicating the model could correctly distinguish all positive (severe) types from negative (non-severe) types. In this study, we run this modeling and predicting process 100 times to evaluate how system stochasticity influenced the RF model and whether the RF model performance was robust. In each of the 100 runs, a different set of randomly selected 80% data were used to train the model and the remaining 20% to test and evaluate the model performance. Note that the 80% training sample would be different in each independent RF runs. We reported maximum, minimum, and median values of performance metrics (accuracy, F1 score, AUC, etc.). Because most performance metrics were not normally distributed, we used median instead of mean value.


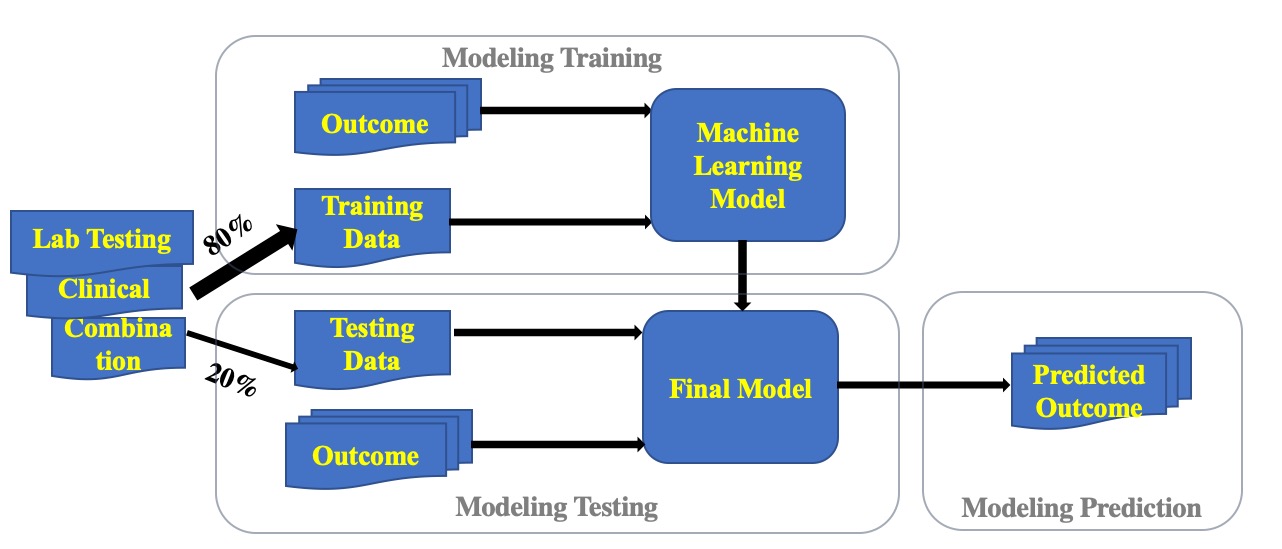


**Fig. S1. Typical Machine Learning Workflow**

**Table S1. Clinical Features and Comparison between Clinical Types**

| **Abbrev.** | **Health Condition/Symptom** | **Gini** | **Coef.** | **OR** | ***p*-value** | **Note** |
| --- | --- | --- | --- | --- | --- | --- |
| **OLD** | Elderly | 24.94 | 1.85 | 13.77  (7.33-25.86) | <0.001 | Age>50 as elderly (OLD=1) |
| **HYP** | Hypertension | 14.43 | 0.63 | 5.37 | <0.001 | Diastolic>90 or systolic>140 |
| **CAR** | Cardiovascular diseases | 8.57 | 0.75 | 5.61  (2.81-11.20) | <0.001 |  |
| **SEX** | Biological gender | 7.79 | 0.63 | 1.90  (1.24-2.90) | 0.003 | Male=0, female=1 |
| **DIA** | Diabetes | 6.73 | 0.39 | 4.61  (2.53-8.38) | <0.001 | Type 2 diabetes only |
| FTG | Fatigue | 6.33 | 0.32 | 1.21  (0.79-1.85) | 0.4 | Subjective, self-reported |
| SHB | Chest congestion | 6.29 | 0.28 | 1.88  (1.22-2.89) | 0.004 |  |
| SOR | Sore throat | 5.92 | -0.9 | 0.30  (0.14-0.61) | <0.001 |  |
| MUC | Phlegm | 5.63 | -0.58 | 0.76  (0.45-1.27) | 0.3 |  |
| FEV | Fever (any) | 5.45 | -0.91 | 1.24  (0.77-1.98) | 0.4 | >37C (>98.6F); measured in the healthcare facility |
| COU | Coughing | 5.41 | -0.05 | 1.00  (0.65-1.54) | 0.9 |  |
| MSA | Muscle ache | 5.39 | -0.58 | 0.78  (0.44-1.36) | 0.4 |  |
| NAP | Loss of appetite | 5.22 | 0.77 | 3.20  (1.70-6.01) | <0.001 |  |
| CON | Contacting COVID-19 patients | 4.33 | -0.28 | 0.71  (0.38-1.33) | 0.3 |  |
| MDF | Medium fever | 4.31 | 1.44 | 1.17  (0.72-1.90) | 0.5 | 38.1-39C (100.5-102.2F) |
| LOF | Low fever | 4.29 | 1.3 | 0.88  (0.56-1.38) | 0.6 | 37.1-38C (98.7-100.4F) |
| CHL | Chilling and shaking | 4.22 | 0.91 | 2.21  (1.16-4.22) | 0.01 |  |
| DIR | Diarrhea | 3.86 | -0.45 | 1.00  (0.53-1.90) | 0.9 |  |
| HIF | High fever | 3.85 | 1.57 | 1.61  (0.88-2.94) | 0.1 | >39C (>102.2F) |
| VOM | Vomiting | 2.79 | -1.7 | 0.34  (0.10-1.26) | 0.1 |  |
| KID | Renal diseases | 1.84 | 1.73 | 8.51  (1.86-38.99) | 0.001 |  |
| HED | Headache | 1.8 | 0.07 | 0.51  (0.23-1.14) | 0.1 | Any type and severity of headache |
| CNC | Cancer | 1.78 | 0.16 | 2.37  (0.90-6.27) | 0.07 | Any type of cancer |
| FAM | Family members with COVID-19 | 1.54 | 1.11 | 1.78  (0.77-4.08) | 0.2 |  |
| SMK | Smoking history | 1.15 | -0.75 | 1.69  (0.60-4.77) | 0.3 |  |
| CPD | Chronic obstructive pulmonary disease (COPD) | 1.08 | 16.28 | N/A | N/A |  |

**Note: bold** are the top five critical clinical features to differentiate COVID-19 non-severe and severe types based on Gini impurity importance score from machine learning random forest model. Logistic regression coefficient signs (positive or negative) reveal if the feature increases or decreases the risk of developing severe type COVID-19. Gini impurity score might not coincide with logistic regression coefficient as they were from two different approaches. OR means odds ratio derived from the 2x2 contingency table with corresponding confidence interval (CI). *p*-values were associated with OR from the contingency table. OR cannot be computed for COPD because no individuals in non-severe type had COPD.

**Table S2. Laboratory Testing Features**

| **Abbrev.** | **Laboratory Testing** | **Gini** | **Coef.** | ***p*-value** | **Unit and Note** |
| --- | --- | --- | --- | --- | --- |
| **DD** | D-dimer | 25.41 | 0.5 | <0.001 | mg/L |
| **hsTNI** | High sensitivity Troponin I | 16.06 | 0.0031 | <0.001 | ng/mL |
| **LDH** | Lactate dehydrogenase | 10.19 | 0.0012 | <0.001 | U/L |
| **NE** | Neutrophil | 10.02 | 0.0044 | <0.001 | 10^9^/L |
| **IL6** | Interleukin 6 | 9.41 | 0.026 | <0.001 | ng/mL |
| hsCRP | High sensitivity C-reactive protein | 9.11 | 0.019 | <0.001 | ug/L |
| ESR | Erythrocyte sedimentation rate | 7.96 | 0.029 | <0.001 | mm/h |
| TBIL | Total bilirubin | 7.43 | 0.018 | <0.001 | umol/L |
| CD8 | Cluster of differentiation 8 | 7.09 | -0.097 | <0.001 | /uL |
| CK | Creatine kinase | 6.7 | 0 | <0.001 | U/L |
| CRP | C-reactive protein | 6.69 | -0.02 | <0.001 | ug/L |
| FER | Ferritin | 5.86 | 0 | <0.001 | ug/L |
| ALT | Alanine transaminase | 5.52 | -0.0037 | 0.008 | U/L |
| CREA | Creatinine | 4.45 | 0.01 | <0.001 | umol/L |
| LY% | Percent of Lymphocyte | 4.4 | 0.91 | 0.002 | % |
| CD3 | Cluster of differentiation 3 | 4.06 | 0.051 | 0.1 | /uL |
| ALB | Albumin | 4.02 | 0.015 | <0.001 | g/L |
| NE% | Percent of Neutrophil | 3.94 | 0.51 | <0.001 | % |
| PLT | Platelet | 3.91 | 0 | 0.6 | 10^9^/L |
| AST | Aspartate aminotransferase | 3.85 | 0.017 | <0.001 | U/L |
| PCT | Procalcitonin | 3.57 | -0.28 | <0.001 | ng/mL |
| CD4 | Cluster of differentiation 4 | 3.51 | -0.056 | 0.3 | /uL |
| LY | Lymphocyte | 3.26 | -0.055 | <0.001 | 10^9^/L |
| WBC | White blood cell | 3.13 | -0.4 | 0.02 | 10^9^/L |
| BNP | Brain natriuretic peptide | 3.07 | 0.0033 | 0.03 | pg/mL |
| HGB | Hemoglobin | 2.98 | -0.024 | 0.7 | g/L |

**Note:** bold are top five laboratory testing features critical to differentiate COVID-19 non-severe and severe types from machine learning random forest (RF) model. *p*-values were derived from two-sided Kolmogorov-Smirnov test between non-severe and severe types.

Table S3. Logistic Regression Prediction Performance

| **Feature** | **Clinical** | **Laboratory Testing** |
| --- | --- | --- |
| **Accuracy%** | 69.44 | 78.62 |
| **Sensitivity%** | 65.22 | 78.65 |
| **Specificity%** | 76.92 | 78.57 |
| **F1 Score%** | 71.07 | 78.61 |
